# Apoptosis-Inducing Activity of Marine Sponge *Haliclona* sp. Extracts Collected from Kosrae in Nonsmall Cell Lung Cancer A549 Cells

**DOI:** 10.1155/2015/717959

**Published:** 2015-07-06

**Authors:** Woori Bae, Hyun Kyung Lim, Kyoung Mee Kim, Hyosun Cho, Sun Yi Lee, Choon-Sik Jeong, Hyi-Seung Lee, Joohee Jung

**Affiliations:** ^1^College of Pharmacy, Duksung Women's University, Seoul 132-714, Republic of Korea; ^2^Innovative Drug Center, Duksung Women's University, Seoul 132-714, Republic of Korea; ^3^Korea Institute of Ocean Science & Technology, Ansan 426-744, Republic of Korea

## Abstract

Although various anticancer drugs have been developed for the treatment of nonsmall cell lung cancer, chemotherapeutic efficacy is still limited. Natural products such as phytochemicals have been screened as novel alternative materials, but alternative funds such as marine bioresources remain largely untapped. Of these resources, marine sponges have undergone the most scrutiny for their biological activities, including antiinflammatory, antiviral, and anticancer properties. However, the biological mechanisms of the activities of these marine sponges are still unclear. We investigated the anticancer activity of marine sponges collected from Kosrae in Micronesia and examined their mechanisms of action using nonsmall cell lung cancer A549 cells as a model system. Of 20 specimens, the *Haliclona* sp. (KO1304-328) showed both dose- and time-dependent cytotoxicity. Further, methanol extracts of *Haliclona* sp. significantly inhibited cell proliferation and cell viability. A549 cells treated with *Haliclona* sp. demonstrated induced expression of c-Jun N-terminal kinase (JNK), p53, p21, caspase-8, and caspase-3. The percentage of apoptotic cells significantly increased in A549 cultures treated with *Haliclona* sp. These results indicate that *Haliclona* sp. induces apoptosis via the JNK-p53 pathway and caspase-8, suggesting that this marine sponge is a good resource for the development of drugs for treatment of nonsmall cell lung cancer.

## 1. Introduction

Complementary medicine has been used for the enhancement of chemotherapeutic efficacy and reduction of adverse effects for several years. Specifically, natural products have been utilized and their active compounds developed as novel anticancer drugs. The major bioresources have largely been phytochemicals, but marine bioresources have been the subject of recent study due to their global abundance. In fact, it has been suggested that more than 3,000,000 such organisms could be good candidates for development of novel drugs. The bioactivities of several biomaterials have been reported, but much remains to be studied. For example, active constituents isolated from soft corals were reported to have anti-inflammatory activity [1, 2]. Additionally, various marine sponges are known to have antibacterial, anti-inflammatory, antiangiogenic, and cytotoxic activities [3–6]. However, the mechanisms of action for these activities are largely unclear. In the present study, we focused on the anticancer effects of marine sponges. In particular, we chose nonsmall cell lung cancer (NSCLC), which has a high mortality due to its resistance to radiation and chemotherapy [7]. Therefore, in an effort to overcome these limitations, we investigated several marine sponge extracts collected from Kosrae, Micronesia, and examined their anticancer activities and mechanisms of action.

## 2. Materials and Methods

### 2.1. Specimen Preparation

Sponge specimens (KO1304 series) were collected by hand with scuba equipment at Kosrae Island in the Federated States of Micronesia in April 2013. The specimens were immediately washed with sterilized artificial seawater and lyophilized. These specimens extracted with methanol (3 × 3 L) were provided by the Korea Institute of Ocean Science & Technology. Each specimen was dissolved in sterile distilled water (final concentration of 50 mg/mL) as previously described [6]. Aliquots of specimens were stored at −20°C until use.

### 2.2. Cells and Treatment

Nonsmall cell lung cancer A549 (CCL-185) cells (ATCC, Manassas, VA) were cultured in Ham's F-12 medium (Gibco, Grand Island, NY) supplemented with 10% fetal bovine serum (GenDEPOT, Barker, TX) and 1% penicillin/streptomycin (GenDEPOT) in a humidified 5% CO_2_ incubator. Cells in the exponential growth phase were used. The samples were added to the medium and treated with extract for 24 h or 48 h.

### 2.3. Cell Cytotoxicity

Cell cytotoxicity was determined using the Cell Counting Kit-8 (CCK-8, DOJINDO, Japan). Briefly, cells (3 × 10^3^ cells/well) were seeded in 96-well plates and incubated for 24 h. After treatment with samples for 24 h or 48 h, CCK-8 reagent (10 *μ*L) was added to each well and incubated for 3 h at 37°C. Absorbance at 450 nm was measured using a microplate reader (Infinite M200 PRO, TECAN, Austria).

### 2.4. Cellular Morphology

Cells (3 × 10^3^ cells/well) were seeded in 96-well plates and incubated for 24 h. Cells were treated with extract for 24 h or 48 h and observed under light microscopy (40x magnification) (Nikon Eclipse TS100, Japan).

### 2.5. Clonogenic Assay

Cells were seeded in six-well plates at a density of 100–500 cells/well. After 24 h, cells were treated with extract and incubated until colony formation. Colonies consisting of at least 50 cells were fixed and stained with crystal violet (0.5% w/v) in 10% methanol and counted. Plate efficiency (PE) and survival fraction (SF) were calculated using the following [8]:

(1) PE=Number of colonies countedNumber of cells plated×100,SF=PE of treated samplePE of control×100.

### 2.6. Western Blot Analysis

Cells were seeded in a six-well plate at a density of 4–6 × 10^4^ cells/well and allowed to incubate for 24 h. Extract was added to each well and incubated for 48 h. Cells were harvested and lysed in RIPA buffer (GenDEPOT) with protease inhibitors (Xpert protease inhibitor cocktail solution, GenDEPOT) and phosphatase inhibitors (Xpert phosphatase inhibitor cocktail solution, GenDEPOT). Cell lysates were boiled in 5x sample buffer and separated by 10% SDS-PAGE. Proteins were transferred onto PVDF membranes (Millipore) using a semidry electroblotter (Peqlab, Germany). Membranes were blocked with 5% skim milk in TBS-T (50 mM Tris-HCl pH 7.4, 150 mM NaCl, 0.1% Tween 20) and sequentially incubated overnight with primary antibodies at 4°C. Membranes underwent additional incubation at room temperature and were then probed with secondary antibody. Immunoreactive proteins were visualized using ECL reagents and developed with X-ray film. Antibodies and dilutions are as follows: p53, p21 (1 : 2000, Millipore), c-Jun N-terminal kinase (JNK, 1 : 500, Santa Cruz Biotechnology), Bax (1 : 1000, Cell Signaling), caspase-3, caspase-8, caspase-9 (1 : 1000, Cell Signaling), *β*-actin (1 : 5000, Sigma-Aldrich), anti-mouse IgG (H+L) horseradish peroxidase conjugate, and anti-rabbit IgG (H+L) horseradish peroxidase conjugate (1 : 3000, Bio-Rad).

### 2.7. Cell Cycle

Cells were seeded in a 60-mm plate and treated with extract (25 *μ*g/mL or 50 *μ*g/mL) for 1 h, 2 h, 4 h, 8 h, 12 h, or 24 h. Incubated cells were collected and fixed with 70% ethanol. Fixed cells were washed with PBS and stained with propidium iodide (PI) staining solution. Cell cycle was detected using a BD FACSCanto II system (BD biosciences, San Jose, CA, USA).

### 2.8. Apoptosis

Cells were seeded in a six-well plate and treated with extract for 24 h or 48 h. Incubated cells were stained with Annexin V-FLUOS staining kit (Roche, Mannheim, Germany). Apoptosis was detected using a Guava easyCyte flow cytometer (Merck Millipore, Darmstadt, Germany).

## 3. Results and Discussion

### 3.1. Screening Test of KO1304 Series

**Figure 1:**
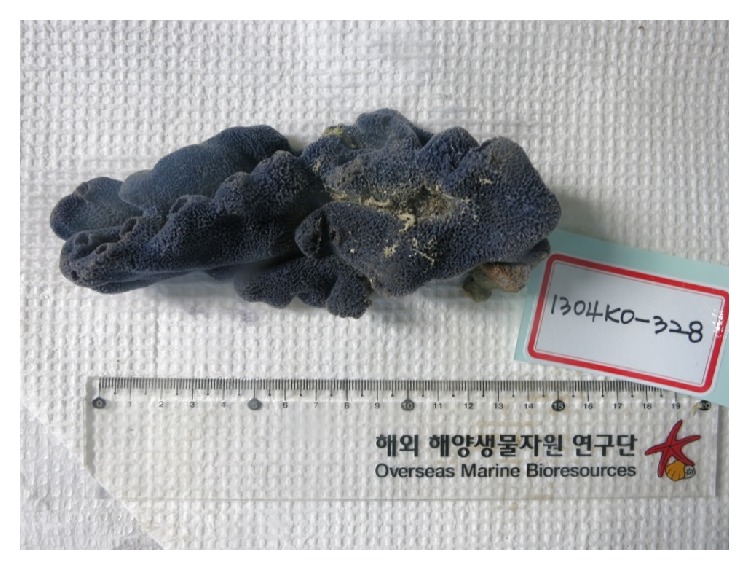
Morphology of* Haliclona* sp.

The cytotoxicity of 20 sponge specimens (KO1304 series) was determined in order to determine their use as an anticancer drug candidate resource. Each specimen was serially diluted and applied to A549 cells for 48 h. As shown in Table 1, despite the inactivity of the other specimens, KO1304-328 inhibited cell viability by more than 30%. KO1304-328 was identified as* Haliclona* sp. (Figure 1), which has been reported to have cytotoxic [9], antibacterial [10, 11], antifungal [11], and anticancer effects in breast, prostate, and colon cancer cells [9, 12]. Papuamine and haliclonadiamine isolated from* Haliclona* sp. were reported as active components [12]. However, the anticancer mechanism of* Haliclona* sp. is unclear, particularly that against human nonsmall cell lung cancer.* Haliclona* sp. extract is widely described in complementary medicine, although the bioactive constituents of* Haliclona* sp. had not been yet isolated and evaluated. Therefore, the mechanism of the anticancer activity of* Haliclona* sp. extract was investigated in human nonsmall cell lung cancer A549 cells.

### 3.2. *Haliclona* sp. Suppresses Cell Viability and Cell Proliferation

**Figure 2:**
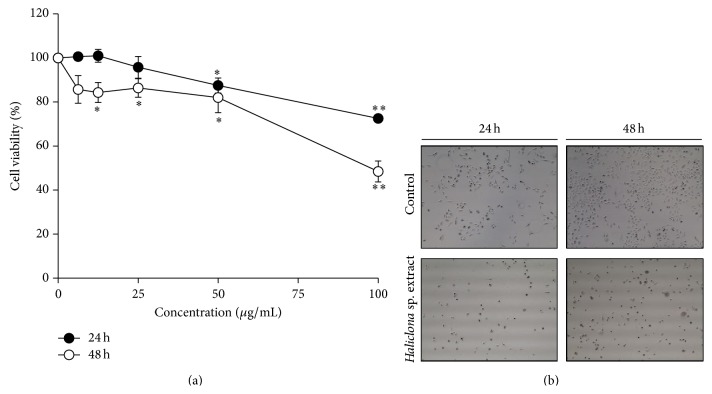
*Haliclona* sp. extract inhibits cell viability in A549 cells. (a) A549 cells were treated with* Haliclona* sp. extract for 24 h or 48 h. (b)* Haliclona* sp. extract (100 *μ*g/mL) was added to the medium. Cells were observed by microscopy (40x magnifications).

To evaluate cytotoxicity,* Haliclona* sp. extract was serially diluted and applied to A549 cells for 24 h or 48 h. As shown in Figure 2(a), cell viability of A549 cells decreased in a dose- and time-dependent fashion. At 24 h, cytotoxicity was statistically significant for extracts of 50 and 100 *μ*g/mL, but maximal cell viability inhibition was only 27.5 ± 2%. In A549 cells treated with* Haliclona* sp. extract for 48 h, a significant difference in cell viability was shown at 12.5, 25, 50, and 100 *μ*g/mL (Figure 2(a)). Further, the maximum dose (100 *μ*g/mL) inhibited cell viability by 51.6 ± 4.7%. Concurrently, we treated A549 cells with a control or* Haliclona* sp. extract and observed the decrease of cell density at 24 or 48 h (Figure 2(b)). Furthermore, the cytotoxicity of* Haliclona* sp. extract showed only in A549 cells, but not in RAW264.7 cells as noncancerous cell line (see Supplemental Data 1 in the Supplementary Material available online at http://dx.doi.org/10.1155/2015/717959). This data suggests that* Haliclona* sp. extract exerts an anticancer effect in a dose- and time-dependent manner.

**Figure 3:**
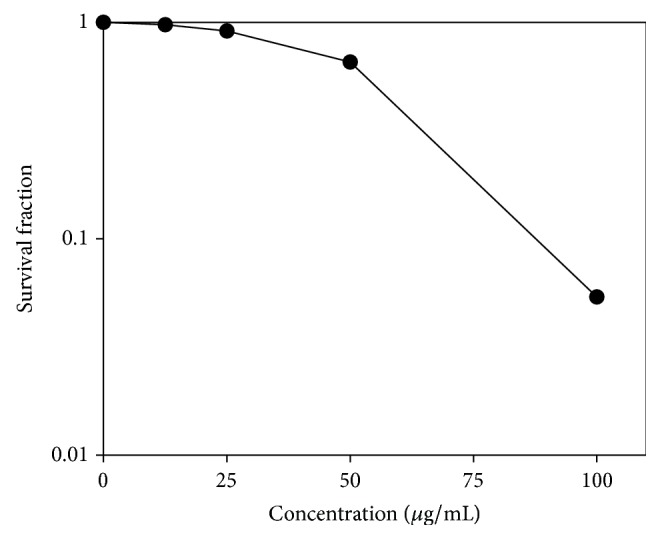
*Haliclona* sp. extract inhibits colony formation in A549 cells. A549 cells were treated with* Haliclona* sp. extract and incubated until the formation of colonies. After two weeks, colonies were counted, and the survival ratio was calculated as described in Section 2.

We also investigated the effect of* Haliclona* sp. extract on cell proliferation. Single, untreated A549 cells proliferated and formed colonies, but those cells treated with* Haliclona* sp. extract were suppressed in colony formation ability (Figure 3). This result indicates that* Haliclona* sp. extract dose-dependently inhibits cell proliferation.

**Figure 4:**
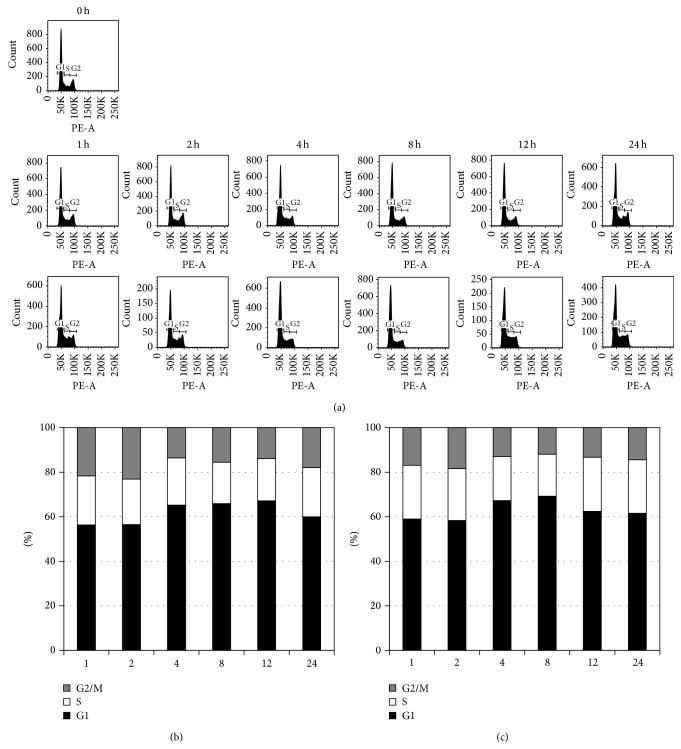
*Haliclona* sp. extract induced cell cycle arrest in A549 cells. (a) DNA histograms of A549 cells treated with* Haliclona* sp. extract (upper, 25 *μ*g/mL; lower, 50 *μ*g/mL). The cell cycle distribution of A549 treated with* Haliclona* sp. extract (25 *μ*g/mL (b) and 50 *μ*g/mL (c)) was analyzed by flow cytometry.

Inhibition of cell proliferation was commonly induced by the cell cycle arrest [13, 14]. Radiation is well known for inducing the permanent G1 arrest and suppressing the cell proliferation [13]. So, we investigated if* Haliclona* sp. extracts affected the cell cycle as radiation. For analysis of cell cycle, A549 cells treated with* Haliclona* sp. extracts were stained with PI solution and detected the phases of the cycle (Figure 4).* Haliclona* sp. showed slight and temporary G1 phase arrest (Figures 4(b) and 4(c)). The results suggested that* Haliclona* sp. could delay the cell proliferation.

### 3.3. *Haliclona* sp. Induces Apoptosis via the JNK and Extrinsic Pathway

**Figure 5:**
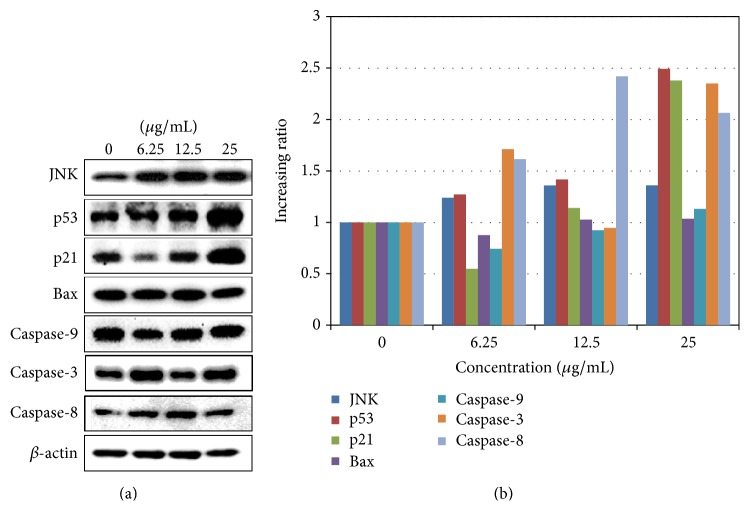
*Haliclona* sp. extract induces the expression of proteins related to the JNK and extrinsic apoptotic pathway. A549 cells were treated with* Haliclona* sp. extract for 48 h. (a) Protein levels were determined using Western blotting. (b) Protein bands were analyzed by ImageJ software. The data were calculated by the ratio of each to *β*-actin bands (as corresponding bands).

To investigate the cellular mechanism of* Haliclona* sp. extract, protein levels were analyzed using Western blots. Specifically, we examined the apoptosis-inducing factors of JNK, p53, Bax, caspase-3, caspase-8, and caspase-9 which activate the mitochondrial or intrinsic apoptotic pathway [15]. JNK phosphorylates and regulates the activity of p53 and its stability [16–18]. The p53 tumor suppressor gene plays an important role in cell cycle, DNA repair, replicative senescence, and cell death [19]. As shown in Figure 5, A549 cells treated with* Haliclona* sp. extracts displayed significantly increased levels of JNK and p53 proteins, as well as p21 a downstream gene upregulated by p53. In several studies, the JNK-p53 pathway was also reported as one of the apoptotic pathways induced by natural products [16, 20]. However, levels of markers of the intrinsic apoptosis pathway, Bax and caspase-9, were not changed by* Haliclona* sp. extracts. Interestingly, the markers of extrinsic apoptosis pathway, caspase-8 and caspase-3, were increased by* Haliclona* sp. extracts. The results suggest that* Haliclona* sp. extracts activate JNK and caspase-8, thereby activating the apoptotic pathway and inhibiting cell viability and proliferation. Still,* Haliclona* sp. extract remained to be determined regarding the upper stream of JNK and caspase-8. Several studies reported the relationship between JNK and caspase-8 induced apoptosis [21–23]. TRAIL death receptor activates caspase-8 and also JNK [21–23]. It may influence the anticancer effect of* Haliclona* sp. extract.

**Figure 6:**
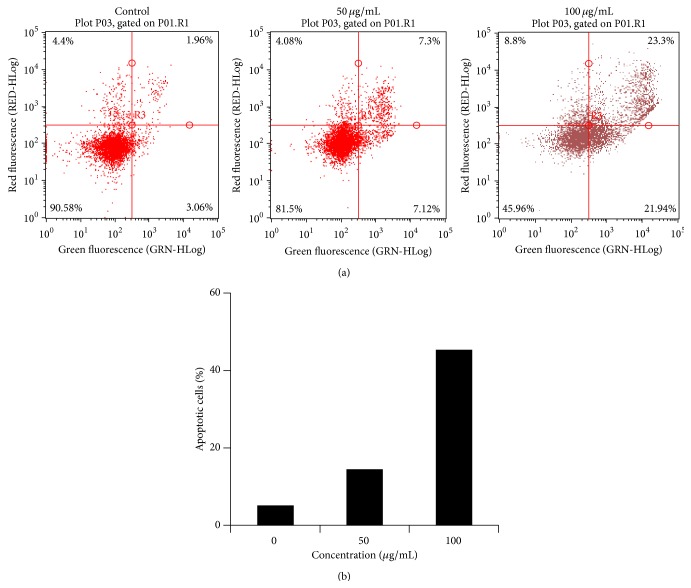
*Haliclona* sp. extract increased apoptosis. A549 cells were treated with* Haliclona* sp. extract for 48 h. Apoptotic cells were determined by Annexin V and PI staining and analyzed as described in Section 2. (a) Upper left, necrosis (%); upper right, late apoptosis (%); lower left, viable (%); lower right: early apoptosis (%). (b) The data showed total apoptosis (early apoptosis and late apoptosis).

To verify the induction of apoptosis, cells treated with* Haliclona* sp. extract were stained with Annexin V and PI. As shown in Figure 6, the percentage of apoptotic cells increased in a dose-dependent manner, suggesting that* Haliclona* sp. extracts have apoptosis-inducing activity via the JNK and extrinsic apoptotic pathway.

## 4. Conclusions

**Figure 7:**
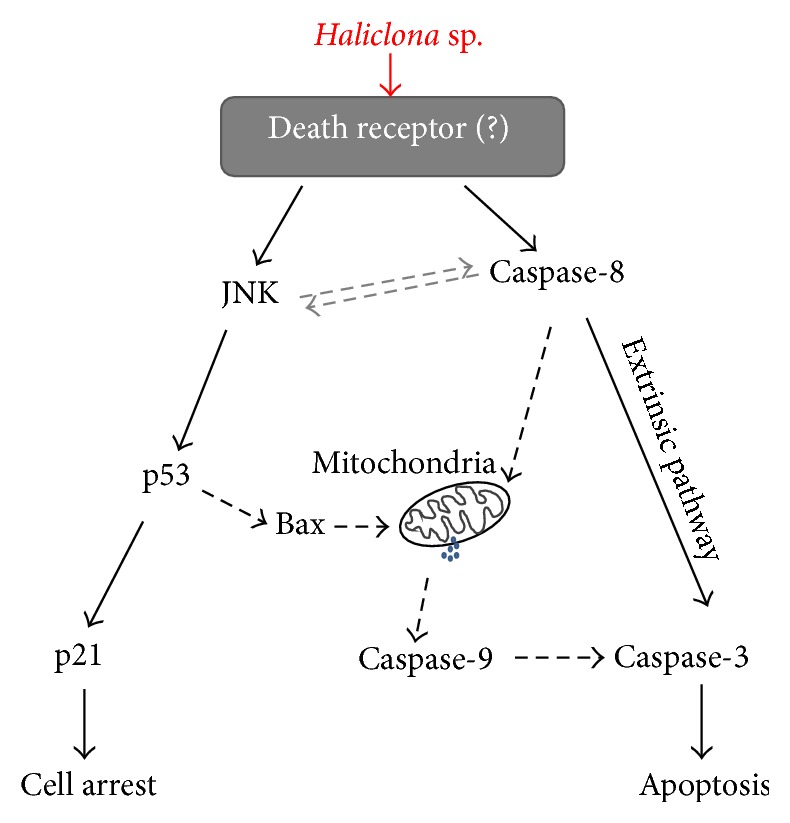
Molecular mechanism of anticancer action induced by* Haliclona* sp. extract.

In this study, we examined the chemotherapeutic effects of marine sponges against A549 nonsmall cell lung cancer cells. We found that a single isolate,* Haliclona* sp., had significant anticancer activity and investigated its mechanism. Our results indicate that* Haliclona* sp. extracts suppress cell viability and proliferation. Eventually,* Haliclona* sp. extract could induce apoptosis* via* activation of JNK and caspase-8 (Figure 7). Thus, the apoptosis-inducing activity of* Haliclona* sp. extract could be utilized for future development of chemotherapeutic drugs against nonsmall cell lung cancer.

## Supplementary Material

Supplemental data 1. Cell viability of Haliclona sp. extract in Raw264.7 cells
Mouse monocyte Raw264.7 cells were seeded in 96-well plate and treated with Haliclona sp. extracts for 24 h or 48 h. Cell viability was determined by cell counting kit-8 assay (n=8). The data showed mean ± standard deviation.

## Figures and Tables

**Table 1:** Cytotoxicity of KO1304 series in A549 cells.

Sample name	IC_30_ (*μ*g/mL)	Sample name	IC_30_ (*μ*g/mL)
KO1304-101	>100	KO1304-207	>100
KO1304-102	>100	KO1304-208	>100
KO1304-103	>100	KO1304-221	>100
KO1304-105	>100	KO1304-223	>100
KO1304-107	>100	KO1304-301	>100
KO1304-201	>100	KO1304-302	>100
KO1304-203	>100	KO1304-303	>100
KO1304-204	>100	KO1304-326	>100
KO1304-205	>100	KO1304-328	68.3 ± 0.4
KO1304-206	>100	KO1304-329	>100

Each extract (titrated from 6.25 to 100 *μ*g/mL) was added to A549 cells for 48 h. IC_30_ is the concentration at which 30% inhibition of cell viability is achieved.
