# Supplementary material for: Apoptosis-Inducing Activity of Marine Sponge Haliclona sp. Extracts Collected from Kosrae in Nonsmall Cell Lung Cancer A549 Cells
Source: Evid Based Complement Alternat Med. 2015 Jul 6;2015:717959. doi: 10.1155/2015/717959 (PMC4508479; doi:10.1155/2015/717959)
Supplement: Supplementary file 1 — Supplemental data 1. Cell viability of Haliclona sp. extract in Raw264.7 cells Mouse monocyte Raw264.7 cells were seeded in 96-well plate and treated with Haliclona sp. extracts for 24 h or 48 h. Cell viability was determined by cell counting kit-8 assay (n=8). The data showed mean ± standard deviation. [file 717959.f1.pptx]

## Slide 1
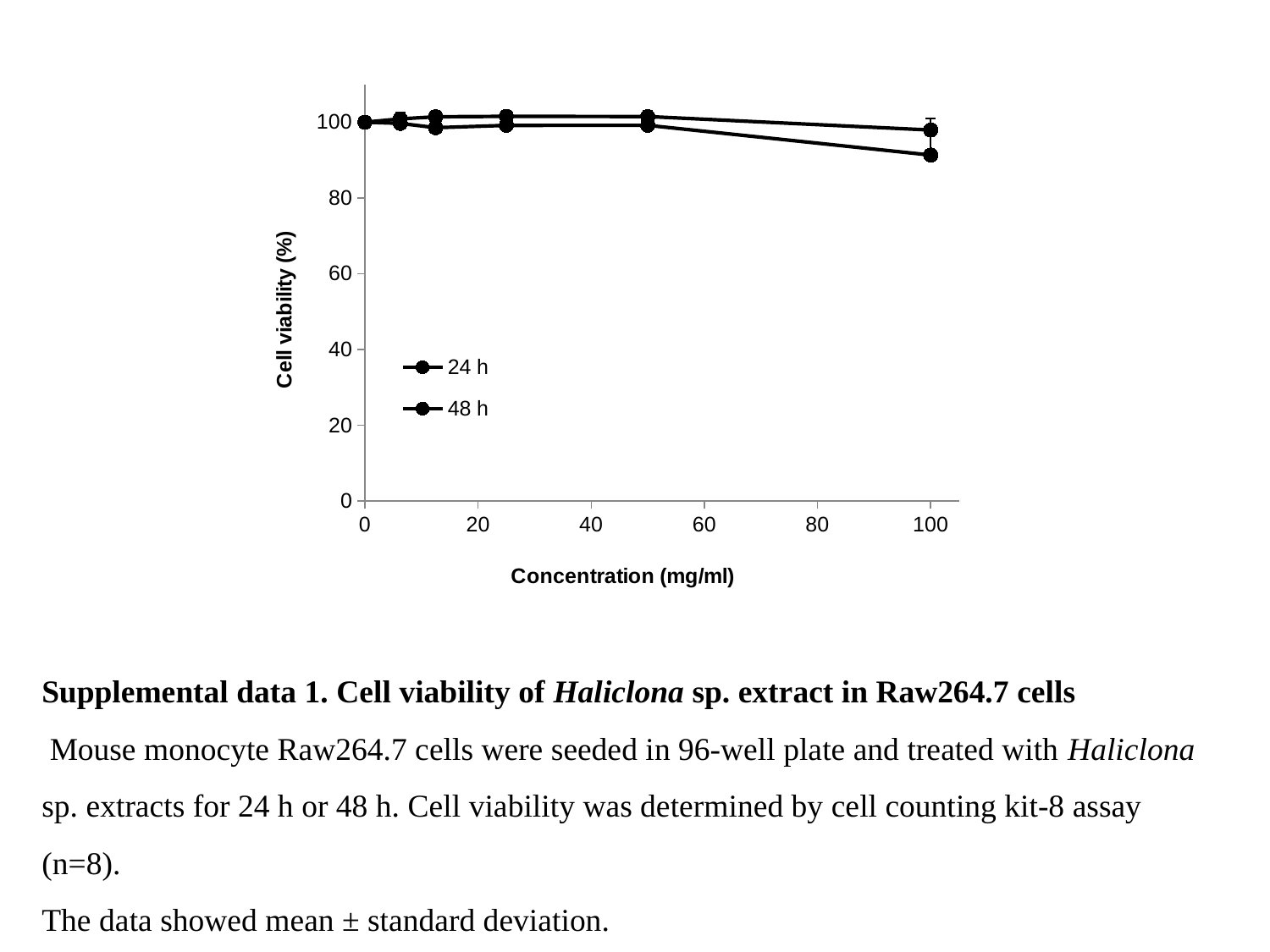

### Chart
| Category | 24 h | 48 h |
|---|---|---|Supplemental data 1. Cell viability of Haliclona sp. extract in Raw264.7 cells
 Mouse monocyte Raw264.7 cells were seeded in 96-well plate and treated with Haliclona sp. extracts for 24 h or 48 h. Cell viability was determined by cell counting kit-8 assay (n=8).
The data showed mean ± standard deviation.
